# Invalid Results in the GetaKit Study in Ottawa: A Real-World Observation of the INSTI® HIV Self-test Among Persons At Risk for HIV

**DOI:** 10.1097/JNC.0000000000000335

**Published:** 2022-04-26

**Authors:** Patrick O'Byrne, Alexandra Musten, Lauren Orser, Cynthia Horvath

**Affiliations:** Patrick O'Byrne, RN-EC, PhD, FAAN, University of Ottawa, School of Nursing, Ottawa, Ontario, Canada. Alexandra Musten, MA, Ontario HIV Treatment Network, Senior Lead, Testing and Clinical Initiatives, Toronto, Ontario, Canada. Lauren Orser, RN, MScN, University of Ottawa, School of Nursing, Ottawa, Ontario, Canada. Cynthia Horvath, RN, MScN, Ottawa Public Health, Sexual Health and Harm Reduction Services, Ottawa, Ontario, Canada.

**Keywords:** HIV, self-testing, invalid results, Canada, real-world observation, Ottawa

## Abstract

HIV self-testing corresponds with more frequent testing, better user satisfaction, and higher positivity rates compared with clinic-based testing. We implemented an open cohort prospective observational study, which provided a website through which persons could do online HIV self-assessments and, if eligible, receive a free HIV self-test. We implemented this project on July 20, 2021 and used the bioLytical INSTI® test. Herein, we describe the number of tests participants reported as invalid, which started at a rate of one fifth of all ordered tests and decreased to 8% after we provided more instructions on completing the test. Our data suggest that a high rate of invalids occur with self-testing in the real-world. Although this has cost implications, we feel this rate is acceptable, considering that 25% of our cohort reported no previous HIV testing. Our take-away message is that HIV self-testing requires additional supports and resources to function as an effective testing intervention.

## Introduction

Despite over 40 years of research and prevention work, in Canada, HIV continues to affect the same groups (referred to herein as “priority populations”), with incidence remaining highest among gay, bisexual, and other men who have sex with men, individuals who are trans, persons of African, Caribbean, or Black ethnicities, members of Indigenous communities, persons from regions where HIV is endemic, and persons who use drugs (Haddad et al., 2021; OHESI, 2019). In many areas of Canada, these groups account for over 95% of new HIV infections per year (Friedman et al., 2017), with an overall estimate that approximately 14% of all persons living with HIV are undiagnosed and unaware of their infection status (PHAC, 2021).

HIV self-testing is one strategy to potentially increase testing among members of HIV priority populations, and research shows that self-testing, compared with clinic-based testing, corresponds with increased testing rates and frequencies, higher positivity rates, and higher satisfaction scores (Edelstein et al., 2020; Johnson et al., 2021; Marks et al., 2021; Menza et al., 2021; Pai et al., 2013; Wachinger et al., 2021; Witzel et al., 2020). Because diagnosis is the first step in the HIV care cascade, technologies that reduce barriers to testing may help achieve the UNAIDS (2021) 95-95-95 targets by decreasing the number of persons with undiagnosed HIV.

One item that could undermine the benefits of self-testing, however, is real-world performance, that is, test performance outside research and clinic settings (Figueroa et al., 2018; Stevens et al., 2018). In addition to ensuring persons use HIV self-tests at the correct timing relative to window periods, test failures (i.e., invalid and false-negative results) are important. Invalid results, specifically, may waste the single opportunity for someone to test; they may also undermine user confidence in the test device and self-testing process. Finally, invalid results have cost implications because they use a resource without any confirmed outcome, and they require the use of a subsequent device or serologic testing when persons choose to retest.

To better understand the real-world outcomes of HIV self-testing in Canada, we implemented the GetaKit pilot to determine uptake and test outcomes. Although we have previously reported on project implementation and our online risk assessment process (O'Byrne et al., 2021a; 2021b), in this article we focus on the invalid test results obtained through the GetaKit study. Specifically, we describe the uptake of testing, results reporting rates, and details about the number, rate, and frequencies of invalid results.

## Methods

GetaKit was a prospective open cohort observational study, in which persons in Ontario, Canada could register on GetaKit.ca, complete an HIV risk self-assessment, and, if eligible based on their self-assessment, order a free HIV self-test to their home or other designated pick-up location. GetaKit was launched in Ottawa, Ontario on July 20, 2020 during the COVID-19 pandemic; from April 1, 2021 to July 16, 2021, eight additional regions in Ontario were added for delivery.

### Test Device and Resources

GetaKit used the bioLytical INSTI® HIV self-test (2021), which was licensed by Health Canada for public sale on November 2, 2020. From the company website, the cost of a kit was $35CAD per device and included a product monograph, with instructions to perform the test, a test disc, three test vials, a lancet, and a bandage. Through GetaKit, participants received additional instructions, a link to an online instructional video, and a workstation to perform the test. Participants also received information on HIV prevention, testing, and care, plus resources for other prevention services, condoms, and lubricant. These additional materials were produced at a cost of $10 per test kit. Shipping cost was $10 per delivery.

**Figure 1.**
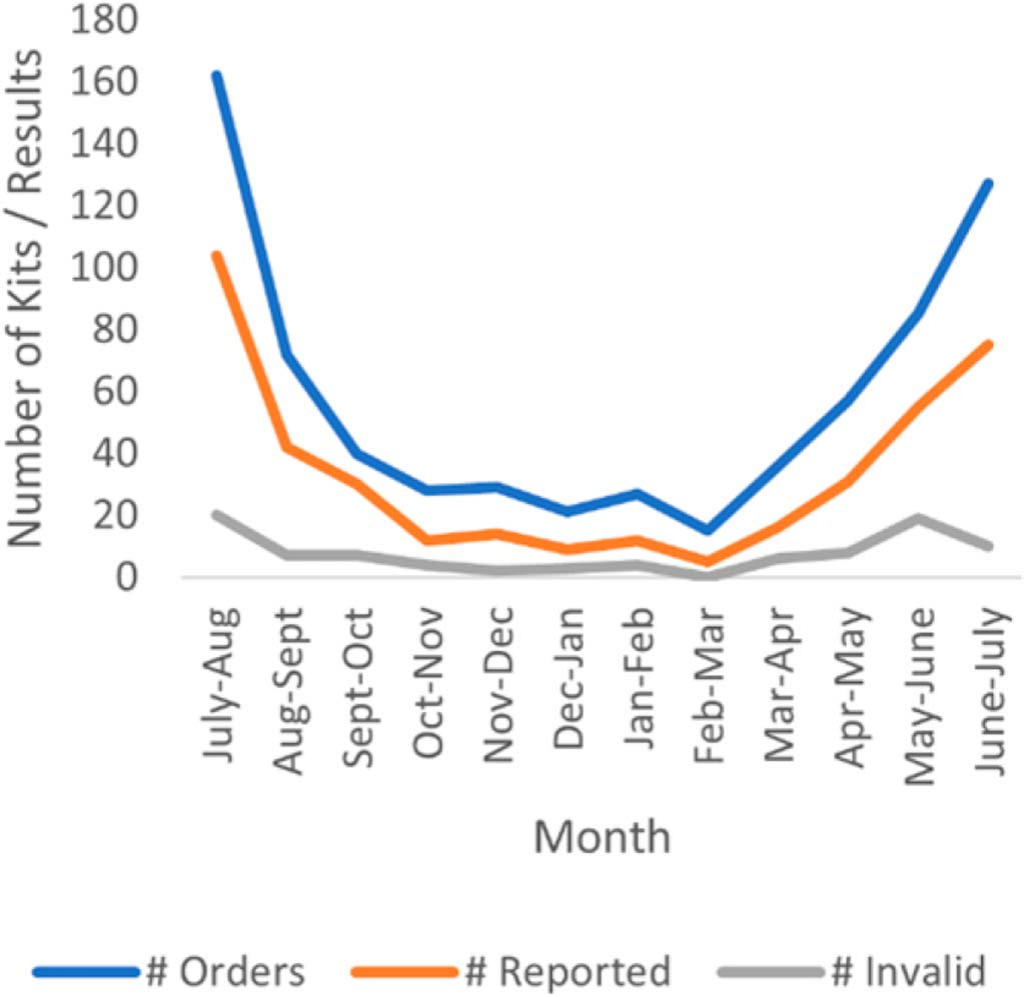
Invalid rates.

### Data Collection

Although participants were not obligated to report their results back via the GetaKit website, they received two reminder messages (at days 10 and 17 from ordering) asking them to do so. Those who wished to reorder a test were, moreover, required to self-report the result of their previous test before they could reorder. Result reporting options were as follows: positive, negative, invalid, and prefer not to report; all results but positive allowed reordering. After a status-neutral approach, the GetaKit study offered relevant resources and services to participants who report results. Those who reported invalid results were encouraged to order a new test and were given information in the form of written text and a 90-second video about how to perform the self-test. Those with negative results were given information about HIV window periods and retesting, about HIV post- and pre-exposure prophylaxis, and about condoms, lubricant, and harm reduction supplies. Those who reported positive results were given support services, confirmatory serologic testing, and referral to an HIV care provider.

Data collection occurred through the GetaKit website, from which all registration, self-assessment, ordering, and results data were exported into an MS Excel file. For this article, the analytic period was anyone who registered within the first year of the project (July 20, 2020 to July 18, 2021), with 24 additional days allotted for results reporting (thereby including the two reminders at days 10 and 17, plus one extra week for result reporting after the final reminder).

### Analysis

For analysis of invalid results, we report on descriptive statistics only. Our interest was to observe the reported rates and if additional strategies would affect these results. Specifically, we analyzed the number of invalid results in relation to the total number of tests ordered and the total number of test results reports. From this, we calculated rates to determine the proportion of all tests that were reported as invalid, proportion of tests with reported results that were marked as invalid, and proportion of ordered tests that were reorders because of a previous invalid result. We did this based on 1-month intervals, starting from July 20 to the 19th of the following month. We also determined if participants obtained repeat invalid results. As a final item, we observed if additional support items decreased the occurrence of invalid test results. In May 2021, immediately when a person ordered a test, we began sending an email with details about how to perform the HIV self-test, including the 90-second instructional video. We reviewed the data to determine if the rate of invalid results changed after we implemented this additional resource.

### Funding and Ethics Approval

GetaKit was funded by the Ontario HIV Treatment Network (EFP-2020-DC1), and ethics approval was obtained from the University of Ottawa Research Ethics Board (H-12-20-6450). All participants provided informed consent to participate in this research.

## Results

From July 20, 2020 to August 19, 2021, 604 participants ordered 701 free at-home HIV self-tests through GetaKit.ca. These participants were, on average, 33 years old. They were primarily White, cis-male, and gay, bisexual, and other men who have sex with men. Additionally, 25% of our participants reported no previous HIV testing, with another 4% being unsure if they had ever completed HIV testing before. Among those who reported previous HIV testing, 45% indicated that it had occurred more than 12 months ago, despite ongoing risk factors for HIV acquisition. See Table 1 for more details.

**Table 1.** Participant Characteristics

	Overall		Invalids		
	#	%	#	%
Ethnicity				
White	397	77	59	82
ACB	99	19	9	13
Indigenous	21	4	4	6
Total	517		71	
Gender				
Cis male	489	70	68	77
Cis female	153	22	13	14
Trans male	15	2	2	2
Trans female	8	1	2	2
Gender nonconforming	32	5	4	4
Total	697		89	
Orientation				
Hetero	137	21	15	20
gbMSM	458	69	54	73
gbWSW	68	10	5	7
Total	663		74	
Tested before				
Yes	490	71	72	79
No	175	25	17	19
Unsure	30	4	2	2
Total	695		91	
Last tested timing				
<12 months ago	258	55	34	50
>12 months ago	207	45	35	50
Total	465		69	

*Note.* ACB = African, Caribbean, or Black ethnicities; gbMSM = gay, bisexual, and other men who have sex with men; gbWSW = gay, bisexual, and other women who have sex with women.

Regarding invalid results, 81 participants reported 89 invalid results, and all but five participants who reported invalid results reordered a self-test. Six participants reported two invalid results, accounting for 13% (*n* = 12/89) of the total number of invalid results reported. All other participants reported subsequent negative test results (57%), did not report back after retesting (14%), or did not reorder a test after receiving an invalid result (29%).

Depending on the period, the reported rate for invalid results varied: for all tests ordered, 0%–22% (average of 12%) of participants reported invalid results; for all reported tests, 0%–38% (average of 22%) of participants reported invalid test results. Excluding the six participants who each reported two invalid results (and the 18 orders they placed), invalid results accounted for 9% of all ordered tests and 12% of all reported results. See Table 2 and Figure 1.

**Table 2.** Test Result Reporting and Invalid Rates

	# Orders	# Reported	# Invalid	% Reported	% Invalid of Ordered	% Invalid of Reported
July–August	162	104	20	64	12	19
August–September	72	42	7	58	10	17
September–October	40	30	7	75	18	23
October–November	28	12	4	43	14	33
November–December	29	14	2	48	7	14
December–January	21	9	3	43	14	33
January–February	27	12	4	44	15	33
February–March	15	5	0	33	0	0
March–April	36	16	6	44	17	38
April–May	57	31	8	54	14	26
May–June	85	55	19	65	22	35
June–July	127	75	10	60	8	13
Total	701	406	89	52	12	22

Finally, after peaking in late May 2021, the invalid rate has since dropped—which corresponds with when we began sending detailed instructions about completing the self-test immediately after participants ordered a test. Table 3 shows this decrease. However, although the number of reported invalid test results decreased, they continued to occur at rates over 10%.

**Table 3.** Invalid Rates Premessaging/Postmessaging Implementation

	# Orders	# Reported	# Invalid	% Invalid of Ordered	% Invalid of Reported
Before emails	529	301	72	14	24
After emails	172	105	17	11	17

## Discussion

Herein, we reported on the invalid results we received during the first year of the GetaKit HIV self-test study in Ontario, Canada. We identified that up to one-fifth of all orders of the INSTI® HIV self-test were reported as invalid, for an average of 12% per month. We also identified that sending instructional information about accurately performing the self-test immediately when participants placed an order corresponded with lower invalid test result rates by 3% overall and 7% for reported results. These findings raise a few points for discussion.

For one, our study identified a higher invalid rate for the INSTI® HIV self-test than what exists in the literature (Galli et al., 2021; Majam et al., 2021). Majam et al. (2021) reported an invalid rate of 0.3% (*n* = 3/900) for this test in their clinic-based study in which participants were observed and evaluated while performing the test. Bwana et al. (2018) reported an invalid rate of 1.26% from their study, in which participants first received a demonstration on how to perform the test in a clinic setting and were then observed performing the test. Galli et al. (2021) reported an invalid rate of 5.6% for the same device, again in a clinic-based study involving direct observation of participants while they performed the test. Our overall invalid rate, in comparison, was 12%, which decreased to 8% after we began providing participants with additional instructions about completing the test.

One interpretation of these data is that, although the INSTI® HIV self-test may be (1) highly sensitive and specific as a device (Bwana et al., 2018; Galli et al., 2021; Majam et al., 2020; Mourez et al., 2018) and (2) easy to use by those who agree to perform the test in a controlled setting while being observed and evaluated, (3) it seems to have lower performance in the real-world when participants use this test alone in a setting of their choosing. It is possible that persons who agree to be evaluated in a research or clinic setting while performing the test are not the same as those who self-test by themselves at home or elsewhere, and that data from controlled settings cannot be extrapolated from observed to unobserved settings. An alternate explanation is that our rate may not be elevated. Further review by of Galli et al.'s (2021) work shows that, in addition to the 5.6% of participants who obtained invalid results, 2.7% experienced difficulty interpreting their HIV self-test results. Taken together, 8.3% (5.6% + 2.7%) of participants in the study by Galli et al. (2021) were therefore unable to obtain a valid/readable test result, which matches our results.

That our final invalid rate matches the overall test failure rate of Galli et al. (2021) suggests that regulators and policy makers should review, approve, and select HIV self-tests based on the combined performance metrics of true test performance (i.e., true invalid results confirmed by a trained observer) plus users' inability to read test results, even if these results would be interpreted as valid by a trained observer. Our results suggest—and align with the work by Galli et al. (2021)—that we might expect an approximately 8% test failure rate for the INSTI® self-test. This finding further suggests that, although HIV self-testing is intended to be done by oneself, additional supports and resources are likely needed to facilitate accurate device usage. Thus, we suggest that nurses who intend to roll out HIV self-testing campaigns using the INSTI® HIV self-test should create resources—e.g., print, video, and other materials—to help persons use these tests so that they work the first time. Such efforts should also include real-time support from nurses, whether virtual, in-person, or by phone, to offer assistance with HIV self-testing, including completing the test and interpreting and managing the results. This is particularly important considering that, for our project, with 89 invalid results and a cost of $40 per kit, we spent $3,560 on testing because of user-defined invalid results. With an invalid rate of 8%, this cost could escalate dramatically depending on volume.

### Limitations

Our findings must be interpreted in light of certain limitations. The study occurred during the COVID-19 pandemic, during a period of great social disruption. The uptake of HIV self-testing, including the characteristics of persons who accessed GetaKit, may have been influenced by the context of a pandemic. It is possible that uptake was higher and/or that more people who would not have used self-testing did so because it was the only available option for care. This may have influenced the invalid rates we observed. This study also took place in a single province (Ontario, Canada), wherein access to HIV confirmatory testing and care is publicly funded. This may have affected participants' perceptions about testing, perhaps making people more willing to test. As well, our observed decrease in invalid rates after adding further instructions was only observed for 2 months afterward. Further evaluation to determine if this decrease will persist is required. Finally, GetaKit being a website likely influenced participation by restricting access to persons with lower computer skills and literacy levels. This may have actually reduced the invalid rate that might be observed among persons with fewer technological skills. Notwithstanding these limitations, this study did identify that real-world participants may not reflect those in more controlled studies of HIV self-testing, which should inform future policy and practice.

## Conclusion

In implementing our real-world prospective HIV self-test study (GetaKit), we identified an elevated rate of reported invalid results compared with previous studies involving participants who completed these tests in clinical settings under strict observation and evaluation protocols. That our reported invalid rate of 8% aligned with the total test failure number from previous research in the same country using the same test, however, suggests that our findings may be a more accurate assessment of the real-world test performance of the INSTI® HIV self-test. This suggests that the INSTI® HIV self-test is a good—but not great—test and that modifications need to be made to support users when they use it. Supplemental instructional materials seem to be required and should likely be delivered before users obtain their test kit. As an overall assessment, however, considering that many of our participants reported no previous HIV testing and risk factors for HIV acquisition, a test performance of 92% is excellent compared with no testing, especially if testing frequency and familiarity increase with ongoing use of self-testing devices. Increasing testing could help address the first step of the UNAIDS 95-95-95 goals to decrease undiagnosed HIV. As such, our conclusion is that this test should continue to be used as a means to promote HIV self-testing, but with the suggestion that additional resources must be developed.

**Key Considerations**

- HIV self-testing is a good strategy to provide access to care for persons at-risk for HIV
- HIV self-testing needs support for appropriate usage to ensure users obtain valid test results
- Resources should be developed to help support persons as they do HIV self-testing.

## Disclosures

The authors report no real or perceived vested interests related to this article that could be construed as a conflict of interest.

## Funding

Support for this project was provided to Patrick O'Byrne by the Ontario HIV Treatment Network (Grant ID: EFP-2020-DC1).

## Author Contributions

P. O'Byrne was responsible for all aspects of this study, from design to implementation, to analysis and write-up. A. Musten was responsible for study initiation and implementation, data analysis, and write-up. L. Orser was responsible for study implementation, data collection and analysis, and write-up. C. Horvath was responsible for study analysis and write-up.
